# One Health Investigation of SARS-CoV-2 in People and Animals on Multiple Mink Farms in Utah

**DOI:** 10.3390/v15010096

**Published:** 2022-12-29

**Authors:** Caitlin M. Cossaboom, Natalie M. Wendling, Nathaniel M. Lewis, Hannah Rettler, Robert R. Harvey, Brian R. Amman, Jonathan S. Towner, Jessica R. Spengler, Robert Erickson, Cindy Burnett, Erin L. Young, Kelly Oakeson, Ann Carpenter, Markus H. Kainulainen, Payel Chatterjee, Mike Flint, Anna Uehara, Yan Li, Jing Zhang, Anna Kelleher, Brian Lynch, Adam C. Retchless, Suxiang Tong, Ausaf Ahmad, Paige Bunkley, Claire Godino, Owen Herzegh, Jan Drobeniuc, Jane Rooney, Dean Taylor, Casey Barton Behravesh

**Affiliations:** 1 National Center for Emerging and Zoonotic Infectious Diseases, Centers for Disease Control and Prevention, Atlanta, GA 30333, USA; 2 Utah Department of Health and Human Services, Salt Lake City, UT 84116, USA; 3 Epidemic Intelligence Service, Centers for Disease Control and Prevention, Atlanta, GA 30333, USA; 4 Centers for Disease Control and Prevention, National Institute for Occupational Safety and Health, Morgantown, WV 26505, USA; 5 Utah Department of Agriculture and Food, Salt Lake City, UT 84129, USA; 6 Utah Public Health Laboratory, Utah Department of Health and Human Services, Salt Lake City, UT 84129, USA; 7 National Center for Immunization and Respiratory Diseases, Centers for Disease Control and Prevention, Atlanta, GA 30333, USA; 8 CDC National Center for HIV/AIDS, Viral Hepatitis, STD and TB Prevention, Centers for Disease Control and Prevention, Atlanta, GA 30333, USA; 9 United States Department of Agriculture, Animal and Plant Health Inspection Service, Veterinary Services, Fort Collins, CO 80526, USA

**Keywords:** SARS-CoV-2, coronavirus, COVID-19, zoonotic transmission, animals, mink, *Neogale vison*, One Health

## Abstract

From July–November 2020, mink (*Neogale vison*) on 12 Utah farms experienced an increase in mortality rates due to confirmed SARS-CoV-2 infection. We conducted epidemiologic investigations on six farms to identify the source of virus introduction, track cross-species transmission, and assess viral evolution. Interviews were conducted and specimens were collected from persons living or working on participating farms and from multiple animal species. Swabs and sera were tested by SARS-CoV-2 real-time reverse transcription polymerase chain reaction (rRT-PCR) and serological assays, respectively. Whole genome sequencing was attempted for specimens with cycle threshold values <30. Evidence of SARS-CoV-2 infection was detected by rRT-PCR or serology in ≥1 person, farmed mink, dog, and/or feral cat on each farm. Sequence analysis showed high similarity between mink and human sequences on corresponding farms. On farms sampled at multiple time points, mink tested rRT-PCR positive up to 16 weeks post-onset of increased mortality. Workers likely introduced SARS-CoV-2 to mink, and mink transmitted SARS-CoV-2 to other animal species; mink-to-human transmission was not identified. Our findings provide critical evidence to support interventions to prevent and manage SARS-CoV-2 in people and animals on mink farms and emphasizes the importance of a One Health approach to address emerging zoonoses.

## 1. Introduction

Severe acute respiratory syndrome coronavirus 2 (SARS-CoV-2), the virus that causes coronavirus disease 2019 (COVID-19), was first identified in Wuhan, China in December 2019 and was declared a global pandemic by the World Health Organization on 11 March 2020 [1]. Since that time, a growing range of susceptible animal species, including companion animals, multiple zoo and aquaria species, wildlife, and production animals including farmed mink, have been identified through both experimental and natural infections. In addition to natural infection, transmission to animals of the same species under experimental or natural conditions has been reported in mink, hamsters, white-tailed deer, cats, ferrets, and deer mice [2,3,4,5,6,7]. Confirmed or suspected transmission from animals to humans has been reported in farmed mink, hamsters, white-tailed deer, and a domestic cat [8,9,10,11]. As the number of naturally infected species with SARS-CoV-2 continues to increase, the establishment of a natural reservoir host in susceptible domestic or wild animals is of growing concern.

In April 2020, The Netherlands reported the first confirmed SARS-CoV-2 infection in farmed mink to the World Organization for Animal Health (WOAH). As of August 2022, SARS-CoV-2 has been confirmed in farmed mink on 474 farms across 12 countries [12]. Farmed mink have garnered global attention due to their high SARS-CoV-2 susceptibility, clinical and subclinical infections, high mortality, and the ability to transmit SARS-CoV-2 to other mink and animal species [13,14,15,16,17]. Denmark identified SARS-CoV-2-positive mink on 290 farms and an estimated 4000 human sequences that contained mink-associated mutations from June to November 2020 [12,18]. Additionally, a novel variant, known as Cluster 5 and shown to be less sensitive to neutralizing antibodies, was identified in specimens from mink and 12 humans on affected Danish farms and surrounding communities [19,20]. In November 2020, the Danish government ordered the depopulation of over 17 million mink; Cluster 5 has not been identified in humans since September 2020 [18,19,20]. These findings, along with the evidence of both zoonotic and anthroponotic transmission [10,21], and the potential for spillover transmission into other species [13,16,22], including susceptible free-ranging wildlife [12,23], have highlighted the importance of in-depth epidemiologic investigations to assess the public and animal health implications of SARS-CoV-2 outbreaks in farmed mink.

On 10 August 2020, Utah Department of Agriculture and Food (UDAF) officials received reports of suspected SARS-CoV-2 infection in mink on two Utah mink farms following substantial die-offs of mink over several days. On 17 August 2020, the United States Department of Agriculture National Veterinary Services Laboratories (USDA-NVSL) reported SARS-CoV-2 confirmation in mink from both farms, marking the first report of confirmed SARS-CoV-2 in farmed mink in the United States (U.S.). Through 5 November 2020, 10 additional Utah farms experienced confirmed outbreaks of SARS-CoV-2 in mink [23]. The on-farm investigations reported herein utilized a One Health approach, which considers the interconnectedness of humans, animals, and their shared environment. In light of documented zoonotic transmission of SARS-CoV-2 on mink farms in Europe, we conducted a study to further investigate the source of initial transmission to the mink, characterize human cases of COVID-19 associated with the affected mink farms, evaluate cross-species transmission potential and duration of shedding to inform quarantine protocols, and identify opportunities for interventions to prevent SARS-CoV-2 transmission among people and animals on participating Utah mink farms.

## 2. Materials and Methods

### 2.1. Epidemiologic Investigation

During 14 August 2020–13 October 2020, a field team from the Centers for Disease Control and Prevention (CDC) and Utah Department of Health and Human Services (UDHHS) conducted interviews in English or Spanish with participants on six farms that agreed to investigations (i.e., consented to on-farm testing of mink and/or humans). Interviews were conducted with persons working or living on-farm, including the operator (i.e., owner or manager) of each farm. For the purposes of this report, farm workers are defined as farm operators, family members of the operators, or hired employees who reported having direct contact with mink. Two farms (A and B) were in the county where SARS-CoV-2 was first confirmed on the farms, and the other four farms (C–F) were in another county located ~90 miles to the north. On-farm interviews collected data on SARS-CoV-2 clinical symptoms, potential exposures (community and work-related), activities with mink requiring prolonged potential work-related exposure (e.g., handling, feeding, cleaning), use of personal protective equipment (PPE), and other occupational data such as the method of commuting to work or additional work arrangements. Farm operators were asked additional questions about clinical illness in mink and COVID-19 preventive interventions used at the farm.

Confirmed human cases of SARS-CoV-2 infection were defined as (1) having a positive SARS-CoV-2 real-time reverse transcription polymerase chain reaction (rRT-PCR) or (2) serological evidence of SARS-CoV-2 exposure with or without reported history of symptoms consistent with COVID-19. The human infectious period was defined as lasting from 2 days before symptom onset to 10 days after symptom onset based on CDC guidance at the time [24].

Utah state COVID-19 surveillance data (i.e., electronic records of symptoms, exposures, and comorbidities for each case), and electronic SARS-CoV-2 testing records including any laboratory evidence of infection, were reviewed to complete case investigations and epidemiologic timelines for each farm. Additionally, the team collaborated with the UDHHS mobile testing team to host two community testing events, one near Farms A and B on 28 August 2020 (22 days and 25 days, respectively, after the start of the outbreaks on those farms), a second near two additional affected, non-participating farms in an adjoining county on 3 September 2020 (36 days, and 15 days, respectively, after the start of the outbreaks on those farms), to attempt to assess whether on-farm cases may have been associated with ongoing SARS-CoV-2 transmission in the community. Each testing event was preceded by purposive door-to-door outreach from bilingual community health workers with local health departments, targeting areas where mink farm workers were reported to live, but without disclosing that the outreach was associated with mink farm outbreaks.

### 2.2. Human Specimen Collection

The field team first collected nasopharyngeal (NP) swabs and blood from participants for SARS-CoV-2 (rRT-PCR) and serology, respectively, after the first sign of illness in mink on each respective farm. Follow-up sample collection was offered ~2 weeks after the initial sample collection on Farms A and B and again ~6 weeks after initial testing at Farm A to enroll participants who had been unavailable for initial testing.

### 2.3. Animal Specimen Collection

Animal work was conducted under the CDC Institutional Animal Care and Use Committee’s approval (protocol 3104BARMULX) and under the approval of, and in collaboration with, UDAF and UDHHS. Detailed methods describing animal sample collection and sampling strategy on each participating farm are included in Appendix A.

### 2.4. Human SARS-CoV-2 Serological Testing

In human specimens, SARS-CoV-2 serological testing via the VITROS Immunodiagnostic Products chemiluminescent immunoassay (CIA), comprising the VITROS anti-SARS-CoV-2 reagent pack and the VITROS anti-SARS-CoV-2 immunoglobulin G (IgG) calibrator [25], was conducted at CDC.

### 2.5. Animal SARS-CoV-2 Serological Testing

In animal specimens, serological response against SARS-CoV-2 was determined using a species-independent assay that detects antibodies binding to the receptor binding domain of the viral spike (S) protein [26,27]. Briefly, sera were exposed to 2 × 10^6^ RAD of gamma irradiation from a cobalt-60 source and heated to +56 °C for 10 min. Quantitation controls (normal human serum spiked with monoclonal antibody dilutions) were included in every run. To establish assay baseline in mink, 32 negative sera were provided by colleagues from Dalhousie University, USDA Agriculture Research Service National Animal Disease Center, and Danielle Adney and Vincent Munster from the Virus Ecology Section of National Institutes of Allergy and Infectious Diseases [28]. Average negative signal was 0.34x no-serum control with standard deviation of 0.104 and maximum signal 0.69. Similarly, baseline reactivity in dogs was established by assaying 150 pre-SARS-CoV-2 emergence sera collected in 2011 in Iowa, U.S., provided by X.J. Meng and Lynn Heffron, Virginia Tech (average 0.33, standard deviation 0.116, maximum 0.76). Hemolyzed specimens were excluded from these analyses. Based on these results and those published earlier for cats [29], the same cut-off as for humans (2.5) was used for all species except for cats (2.8). Upon finding strong reactivity in mink sera causing assay saturation and hook effect, the specimens were also tested at 1/50 dilution in 1% BSA/PBS. The results from the 1/50 diluted specimens were multiplied by the median undiluted/diluted ratio of non-saturated positive specimens to extrapolate values for mink specimens. Results for other species represent the undiluted sera results as previously established.

CDC also performed SARS-CoV-2 microneutralization experiments on the animal sera using a mNeonGreen reporter virus provided by Pei-Yong Shi, University of Texas Medical Branch) [30]. Vero CCL-81 cells were seeded at 3 × 10^4^ cells/96-well in DMEM with 10% heat-inactivated FCS (HI-FCS; ThermoFisher). A seven-point, three-fold dilution series of the sera (heat-inactivated at 56 °C for 60 min) were prepared in 2% HI-FCS/DMEM so that the first dilution was 1:5. An equal volume of 1.2 × 10^5^ pfu/mL virus in 2% HI-FCS/DMEM was then added to specimen dilutions and the mixes incubated at 37 °C for 30 min. Triplicate wells were infected by adding 50 µL of the mixes (resulting in MOI 0.1 and serum dilutions from 1:10 to 1:7290). The following day, the medium in the wells was replaced to reduce background fluorescence and the wells imaged using a Cytation 3 instrument (BioTek Instruments, Inc., Winooski, VT, USA). Total fluorescent areas were corrected by reducing the value obtained from non-infected wells. Percentage inhibition was calculated using wells infected in the absence of serum sample as the maximal value. The 50% inhibitory dilution (ID50) value was determined using the log(inhibitor) vs. normalized response function of GraphPad Prism software package. Additionally, USDA-NVSL performed conventional SARS-CoV-2 virus neutralization on aliquots of the same sera specimens, as previously described [31,32].

### 2.6. Human Specimen RNA Extraction and SARS-CoV-2 rRT-PCR

For human NP swab specimens, nucleic acid was extracted and SARS-CoV-2 rRT-PCR testing was conducted at Utah Public Health Laboratory following published methods [33]. Equivocal rRT-PCR results were defined as positive results for only 1 of the 3 assay targets, with negative or equivocal results upon repeat testing of the primary specimen.

### 2.7. Animal Specimen RNA Extraction, SARS-CoV-2 rRT-PCR, and Virus Isolation

For animal specimens, the nucleic acid extractions were performed with the Kingfisher Flex Instrument (Thermo Fisher Inc., Waltham, MA, USA), using the MagMAX™ Viral/Pathogen Nucleic Acid Isolation Kit (Thermo Fisher Inc., Waltham, MA, USA), according to the manufacturers’ instructions. The MVP_Flex_200 ul protocol was used with 200 µL specimen volume and an elution volume of 100 µL. Human specimen control (HSC; A549 cell suspension) was included as an extraction control and water as a negative control.

rRT-PCR testing of animal specimens for SARS-CoV-2 was performed on the ABI 7500 Fast Dx Real-time PCR system (Thermo Fisher Inc., Waltham, MA, USA) using the CDC influenza SARS-CoV-2 (FluSC2) multiplex assay (https://www.fda.gov/media/139743/download; accessed on 1 November 2022) and TaqPath™ 1-Step Multiplex Master Mix (No ROX) (Thermo Fisher Scientific Inc., Waltham, MA, USA). rRT-PCR negative specimens were further tested for β-Actin using the Taq polymerase enzyme mentioned above. An animal was classified as “rRT-PCR positive” if specimens from the animal tested positive by at least one diagnostic specimen (oropharyngeal swab, nasal swab, or rectal swab).

Virus isolation was performed by USDA-NVSL as previously described [34].

### 2.8. Genomic Sequencing and Analysis

At Utah Public Health Laboratory (Salt Lake City, UT, USA), residual nucleic acid extractions from rRT-PCR positive human specimens were used for input for the ARTIC amplicon sequencing protocol (Josh Quick 2020. nCoV-2019 sequencing protocol. protocols.io dx.doi.org/10.17504/protocols.io.bdp7i5rn). The amplicons generated were then sequenced on the Illumina MiSeq platform. Consensus sequences were generated using UPHL’s custom analysis workflow Cecret (https://github.com/UPHL-BioNGS/Cecret; accessed on 1 November 2022).

At CDC, nucleic acid from animal specimens with cycle threshold values ≤30 was extracted and subjected to Illumina sequencing following previously published protocols [35]. Consensus sequences were generated with IRMA [36]. Phylogenetic relations of the study sequences were inferred using maximum likelihood analyses implemented in Treetime using the NextStrain pipeline [37]. Randomly selected SARS-CoV-2 sequences from humans in Utah with collection dates through 31 January 2021, and all mink/mink-outbreak-related SARS-CoV-2 sequences were downloaded from the GISAID repository on 3 May 2022, and used for comparison.

### 2.9. Community Engagement and Outreach

In June 2020, CDC, USDA, and other federal and state partners developed recommendations for preventing SARS-CoV-2 introduction and transmission on mink farms [38] to engage mink farms and their surrounding communities in response to the initial identification of SARS-CoV-2 in farmed mink in Europe in April 2020.

After SARS-CoV-2 was identified in farmed mink in the United States in August 2020, prevention recommendations and basic transmission prevention training were provided during and following each of the on-farm investigations from August–November 2020. A seminar entitled “Steps to Prevent COVID-19 on Mink Farms” featuring recommendations and guidance for mink farms was coordinated by public health officials for mink farmers at a Utah Fur Breeders meeting on 3 November 2020, and disseminated via a nationwide webinar on 4 November 2020.

## 3. Results

### 3.1. Epidemiologic Investigation and Human Testing

Among 12 Utah farms with confirmed SARS-CoV-2 in their mink herds, six (50%) actively participated in epidemiologic investigations. Prior to the SARS-CoV-2 outbreaks, each farm had herds of 300–13,500 mink. Estimated losses due to SARS-CoV-2 ranged from ~4 to 50% (Table 1). All participating farms had family residences on-farm where farm operators and/or their family members lived. All were operated independently, with few, if any, hired employees and no shared workers between the six farms. Sharing of equipment did not occur between farms during the two weeks preceding onset of clinical signs. Additionally, no animals were transported on or off the farms during the same period. Mink were housed in roofed sheds with open side walls and two to nine rows of metal cages down the length of the shed. After weaning, mink were housed either individually or with a littermate or dam/kit pair. The participating farms vaccinated their mink annually with a mink enteritis vaccine (trade name Biocom-P, United Vaccines, Inc, Flitchburg, Wisconsin) to protect against *Clostridium botulinum* Type C, parvovirus (mink enteritis), and *Pseudomonas aeruginosa*. Bacterial pneumonia was initially suspected to be the cause of, or contributor to, illness in mink on the initial farms because both farms had a history of morbidity and mortality due to bacterial pneumonia (primarily due to *Psuedomonas* sp.). Most of the affected farms had a history of Aleutian disease virus (ADV) and performed annual screening of their herds. Results and analysis of bacterial testing and ADV antibody testing in investigations on Farms A and B are published separately [26].

Among 17 persons associated with the six participating farms (including farm workers as well as family members living on-farm without reported contact with mink), 16 (94%) persons in total (median per farm: 2.5 persons, range: 1–4 persons) participated; the 1 non-participant did not consent to testing and was not included. NP swabs were collected from all 16 (100%) participants and underwent SARS-CoV-2 rRT-PCR testing; most participants (15/16, 94%) had blood drawn for serological testing by anti-SARS-CoV-2 CIA. SARS-CoV-2 rRT-PCR was positive on NP swabs from 11 of 16 (69%) participants; 9 of these 11 (82%) participants reported clinical symptoms consistent with COVID-19. Among these nine with known symptom onset dates, SARS-CoV-2 RNA was first detected a median of 13 days (range 3–22 days) after symptom onset. Among 15 participants with blood specimens collected, 11 (73%) were SARS-CoV-2 seropositive, including one asymptomatic participant who tested rRT-PCR negative, resulting in 12 human COVID-19 cases confirmed by PCR and/or serology. Specimens were collected at ≥2 TPs from six confirmed cases; intervals between specimen collections (either rRT-PCR or serological) ranged from 5–50 days. The longest duration of rRT-PCR positivity observed was 63 days in a male worker in his 50s. A summary of symptom onset date, diagnostic testing results, and dates of contact with mink for each participant, relative to onset of clinical signs in mink on each farm, is presented in Figure S1.

The 12 confirmed human COVID-19 cases identified by rRT-PCR or serology included ≥1 person from each farm who lived or worked on-farm; 11 (92%) were farm workers and 1 person was an on-farm family member. Among the 12 confirmed cases, 9 (75%) persons across five farms reported symptoms consistent with COVID-19, and 3 (25%) persons across three farms were asymptomatic. The most common symptoms reported were respiratory symptoms (six participants, 67%), loss of smell (four, 44%), loss of taste (three, 33%), and sore throat (three, 33%). Among the 4 out of 16 (25%) persons tested with no laboratory-confirmed evidence (i.e., neither rRT-PCR nor serological evidence) of SARS-CoV-2 infection, one reported having a recent history of symptoms consistent with COVID-19.

The nine farm workers with known symptom onset dates worked with mink a median of two days (range: 1–3 days) during their infectious period (2 days before and ≤10 days after their symptom onset), before or on the first day of increased mortality in mink (Figure S1). The most common work tasks included cleaning mink cages, feeding mink via feed delivery systems, and verifying that water lines were operating correctly. All these activities put workers within six feet (2 m) of mink, typically for ≥15 min, and within three feet (1 m) of mink for feeding and water line checks. The median interval from first exposure of mink to a presumably infectious human case and first sign of illness in mink was two days (range: 1–5 days). On three of the six (50%) participating farms, signs of illness in mink, primarily coughing and decreased appetite, preceded increased mink mortality by a median of three days (range: 1–3 days). At the other three farms, increased mortality was the first reported indication of illness in mink. Among the five farms with known symptom onset in a human case, onset in the first human case preceded the first indication of illness in mink by a median of one day (range: 0–5 days). Among the six farms, increased mortality in mink lasted a median 10 days (range: 6–18 days) and did not appear to vary in length or intensity based on the total number of human cases or the occurrence of new on-farm human cases during the increased mortality period. 

The two community testing events offered no-cost rRT-PCR SARS-CoV-2 rRT-PCR testing to people living in local communities in proximity to affected mink farms. One event, near two of the six participating farms, was held on 28 August 2020. During this event, out of ~75 persons within ~20 households that received door-to-door outreach, 15 persons were tested. The second event, near two additional non-participating farms in an adjoining county, was held on 3 September, and tested 10 additional persons following neighborhood flyer distribution by the local health department. All persons tested received negative SARS-CoV-2 rRT-PCR results.

#### Detailed Epidemiologic Timelines for Farms A and B

**Figure 1:**
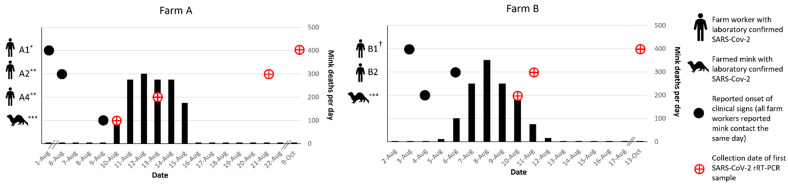
Timeline of symptom onset and SARS-CoV-2 laboratory confirmation in farm workers and in mink relative to onset of increased mortality in mink on Farms A and B. * A1 left work early on 1 August with symptoms but was not tested for SARS-CoV-2 until 9 October (tested positive). ** A2 and A4 both had symptomatic household contacts that tested SARS-CoV-2-positive on 10 and 13 August, respectively. A2 would remain asymptomatic, but ultimately tested positive on 21 August. *** Reported clinical signs in mink included non-specific (e.g., inappetence, lethargy) and respiratory (e.g., sneezing, coughing, nasal and ocular discharge, labored breathing) symptoms. ^†^ B1 symptoms resolved on 9 August. NP swab was collected from B1 on 23 August, and the result on SARS-CoV-2 rRT-PCR was indeterminant. B1′s first positive result was not until 13 October.

Epidemiologic timelines of farm worker illnesses relative to clinical disease course in mink were constructed for the initial two farms, A and B, that participated in longitudinal investigations (Figure 1).

On 1 August, a worker (A1) on Farm A, with a herd of approximately 7000 mink, left work early with complaints of feeling ill (Figure 1). Prior to leaving for the day, this worker fed mink in only one (shed 0) of eight mink sheds and a second worker (A2) completed feeding in the seven remaining sheds. On 6 August, a large number of mink in shed 0 were reported to have inappetence; on the same day, A2 developed a temperature of 101 °F and a dry, non-productive cough. On 10 August, a household contact of A2 tested positive for SARS-CoV-2 and mortality in the mink herd began increasing, with the loss of 100 majority adult female mink in shed 0. The baseline expected level of mortality in August on Farm A was reported to be two to three mink losses per day. On 13 August, another farmworker (A4) and their household contact (A3) were tested for SARS-CoV-2 due to exposure to a separate household contact who was feeling ill in the preceding days. Both A4 and A3 tested positive, though A4 remained asymptomatic. The high mortality rate in mink spread throughout all eight sheds and increased to hundreds of mink deaths per day (Figure 1). In total, Farm A lost more than 1400 (~19.9%) mink by the end of August (Table 1). The greatest proportion of total losses were among adult (>1 year) females with 66.2% loss (1211/1830), followed by adult males with 59.0% loss (171/290), and juveniles (<1 year) with 1.4% loss (72/~5200). A2, A3, and A4 were tested as part of the field investigation on August 21. All tested positive on NP swab by SARS-CoV-2 rRT-PCR and their blood specimens were positive for SARS-CoV-2 antibodies (Figure 1). A1 was tested for the first time on 9 October; both NP swab and sera indicated SARS-CoV-2 infection. Farm A is surrounded by a locked perimeter fence, in which one farm dog and a large feral/free-roaming cat colony reside. Wildlife are seen occasionally within the perimeter fence and are removed by farm workers on identification.

On 3 August, the index patient (B1) on Farm B, which houses a herd of approximately 8000 mink, developed symptoms consistent with COVID-19. B1 reported working on the farm daily for the four days prior to and the day of symptom onset, and then did not return to working on the farm until after symptoms resolved on 9 August. On 4 August, mink in the herd were noted by another farm worker (B2) to have inappetence and respiratory signs. On 6 August, B2, who is also a household contact of the index patient on this farm, developed respiratory symptoms and profound fatigue. On the same day, increased mortality began in the herd, beginning with deaths of 10 adult female mink; the baseline expected level of mortality on Farm B was reported to be one to two mink losses per day. Over the next 8 days, the losses increased to hundreds of mink per day, peaking on 9 August, and declining until returning to baseline by 14 August. In total, Farm B lost 1251 (~15.2%) mink by the end of August (Table 1). The greatest proportion of total losses were among adult (>1 year) females with 49% loss (1127/2298), followed by adult males with 57.2% loss (147/257), and juveniles (<1 year) with 3.1% loss (177/5683). B2 initially tested positive for SARS-CoV-2 on 11 August. B1 was not tested by rRT-PCR until the field investigation began on 25 August at which time the results were reported as indeterminate (one of two targets were detected by SARS-CoV-2 rRT-PCR). Blood specimens collected from B1 and B2 on 25 August were positive for SARS-CoV-2 antibodies. B1 would later test positive for SARS-CoV-2 by rRT-PCR for the first time on specimens collected on 13 October (Figure 1 and Figure S1). Farm B is surrounded by a locked perimeter fence, in which four farm dogs reside. Wildlife and cats were rarely seen within the perimeter fence due to canine exclusion.

### 3.2. Animal Testing

#### 3.2.1. Serological Testing

**Figure 2:**
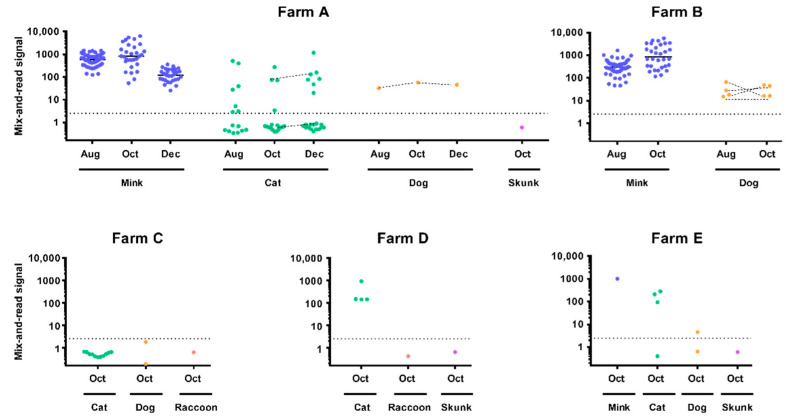
Animal serology. Results of the mix-and-read assay are presented for each tested farm, species, and time-point. The dotted line indicates assay cut-off (2.5; 2.8 used for cats). Connected dots represent re-sampling of the same animal. Mink values are derived from diluted sample specimen results as described in methods. For mink, means of log-transformed values are shown by horizontal lines.

Serological testing was conducted on a total of 212 mink, 15 dog, 72 cat, 2 raccoon, and 3 skunk serum specimens across five farms (Farms A, B, C, D, and E) during the field investigations in August, October, and December 2020 (Figure 2, Table 2 and Table 3).

Longitudinal sampling of the mink herds on Farms A and B yielded 100% seropositivity for SARS-CoV-2 at TP 1 and TP2 with strong overall reactivity. Longitudinal sampling of the cat colony on Farm A yielded 53 total specimens collected from TP1–TP3. The cats were not microchipped for TP1 in August, so it was not possible to know if individual cats from TP1 were resampled at subsequent TPs; however, two cats were resampled between TP2 and TP3. At TP1, specimens from 7 of 15 (46.7%) cats were seropositive. At TP2, specimens from 4 of 17 (23.5%) cats were seropositive. At TP3, specimens from 7 of 21 (33.3%) cats were positive. Of the two cats that were resampled between TP2 and TP3, one cat remained seronegative and one cat remained seropositive at both TPs. Percent seropositivity of 21 cats sampled across Farms C, D, and E ranged from 0 to 100%. Qualitative serology results on a subset of cats from Farms A, C, and D that were affixed with GPS collars during the October TP are presented elsewhere [29].

Longitudinal blood collection for SARS-CoV-2 serology was conducted in one asymptomatic dog on Farm A across three TPs and four asymptomatic dogs on Farm B across two TPs. The dog on Farm A maintained seropositivity at all three TPs; the four dogs on Farm B were also all seropositive at both TPs (Table 2). Asymptomatic dogs on two additional farms were sampled only once in October: zero of two dogs on Farm C and one of two dogs on Farm E was seropositive with low reactivity (Table 3). All specimens collected from wildlife trapped on three farms, including two raccoons and one skunk, were seronegative. Serology results obtained with the mix-and-read assay were corroborated using two different virus neutralization assays conducted in two laboratories. Results from all three assays were in high agreement (Figure S2).

#### 3.2.2. Molecular Testing

Longitudinal sampling on two farms, Farms A and B, at three and two TPs, respectively, revealed prolonged rRT-PCR positivity in mink. Mink on Farm A had similar rRT-PCR positivity at TP1 (67%) and TP2 (70%); at TP3, rRT-PCR positivity decreased to 2.9%. rRT-PCR positivity in mink on Farm B increased from TP1 (52%) to TP2 (100%) (Table 2). On Farms A and B, clinical signs (respiratory or non-specific signs such as inappetence) possibly attributable to SARS-CoV-2 were noted in sampled mink at TP1 and TP2, though herd mortality rate had returned to baseline levels by mid-August. On Farm A, a colony of feral/free-roaming domestic cats and one dog were sampled at three TPs. All cats sampled on Farm A in TP3 were rRT-PCR negative, suggesting that SARS-CoV-2 was likely not circulating in the cat colony by December. The dog on Farm A was rRT-PCR positive at TP1 and negative at subsequent TPs (Table 2).

Clinical signs and increased mortality in mink on Farm C had resolved by the time the field team arrived; no additional mink were sampled on Farm C during field investigations. Clinical signs and herd mortality had improved on Farms D and E at the time of the field investigations, but daily losses remained above baseline on both farms. Farm F, which had the latest onset of clinical signs in the mink herd, was experiencing widespread clinical signs and increased mortality at the time of the investigation. On Farm E, 29 (93.5%) of sampled mink were rRT-PCR positive. Sampled mink included 24 fresh dead mink (23 [95.8%] rRT-PCR positive), 5 mink that had been stored frozen at ~−20°C for >48 h and then thawed completely at room temperature prior to sample collection (100% rRT-PCR positive), one trapped escaped mink without clinical signs (rRT-PCR negative), and one euthanized mink that was critically ill (rRT-PCR positive). On Farm C, one of two dogs was rRT-PCR positive, while none of the eleven cats tested positive. On Farms D and E, one (20%) and four (57%) cats tested rRT-PCR positive. During our investigations in October, a subset of the sampled cats on Farms A, C, and D was also affixed with GPS collars to monitor cat movement patterns on- and off-farm; the findings of this sub-study are published separately [29].

In August, fur swabs from 55 (98.2%) mink on Farm A and 47 (85.5%) mink on Farm B were positive, suggesting extensive environmental contamination within the mink sheds. On Farm A, fur swabs from 6 (37.5%) and 2 (10.5%) cats were positive in August and October, respectively. Fur swabs from 1 (20%) cat on Farms D and E were positive; fur swabs from all 11 cats on Farm C were negative. On Farm A, fur swabs from the dog were negative at both TPs; on Farm B, fur swabs from one (25%) dog were positive at both TPs. On Farm E, fur swabs from one (50%) dog were positive; fur swabs from both dogs at Farm C were negative.

All wildlife trapped and from which specimens were collected during these investigations were rRT-PCR negative on fur swabs and diagnostic specimens, and include house mice (*Mus musculus*) (N = 22 on Farm A and N = 4 on Farm B), one raccoon (*Procyon lotor*) on Farm E; one raccoon and one skunk (*Mephitis mephitis*) on Farm C; and one skunk on Farm D.

#### 3.2.3. Viral Culture

A total of 16 viral isolates were obtained from OP or NP swabs collected from 11 mink on four participating farms (A, D, E, and F). On Farm A, three isolates were obtained from mink in TP1 and two isolates were obtained from one mink (NP and OP) in TP2. One isolate was also obtained from mink on each of Farms D and F. Additionally, isolates were also obtained from both OP and NP swabs from four mink, and OP only from one mink (nine isolates from five animals) on Farm E that had been frozen at approximately −20C for >48 h and then thawed completely prior to specimen collection. No isolates were obtained from specimens obtained from mink at the third TPs, nor from non-mink species.

### 3.3. Genomic Sequencing and Analysis

**Figure 3:**
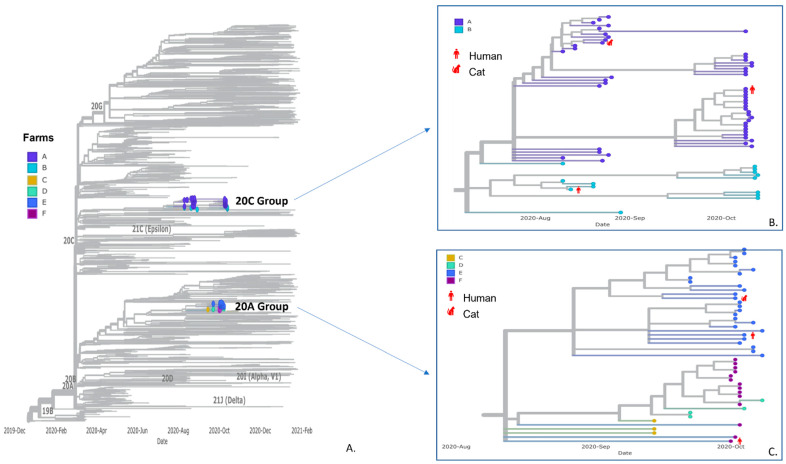
Panel (**A**) SARS-CoV-2 phylogenetic tree including 2250 randomly sampled sequences from human specimens in Utah and sequences from this study. Farm sequences fell onto two branches with zoom-in view of clades 20C group (Panel (**B**); Farms A and B) and 20A group (Panel (**C**); Farms C–F). Sequences are color-coded by farms of origin, and sequences from non-mink hosts are labeled in red with host species.

Whole-genome sequencing generated 113 SARS-CoV-2 sequences from mink, human, and cat specimens from the six different farms, including some previously published sequences [39] (Table S1). These sequences were included in a phylogenetic analysis alongside 2250 selected Utah human sequences, including 407 community sequences from the same counties as the farms. All 113 sequences fell in the B.1 PANGO lineages where sequences from Farms A and B formed a monophyletic group within clade 20C, while sequences from Farms C–F formed a monophyletic group in clade 20A (Figure 3). Sequence comparisons within farms revealed 0–9 SNPs between pairs of sequences (Table 4). Notably, SARS-CoV-2 sequences from non-mink hosts were almost identical to at least one sequence from mink on the same farm, differing by 0-2 SNPs (Figure 3). Some sequences in this study contained Spike protein mutations previously detected in the human population, as well as the Spike protein mutation Y453F, one of the mink-associated Cluster 5 mutations. Sequences at farms with longitudinal sampling across two-month time points (Farms A and B) had a broader range in overall SNP differences, although most of the sequences were still identical or varied by a couple of SNPs (Table 4). Overall, sequences were more likely to cluster within farms as opposed to across farms, even across timepoints, suggesting that transmission had occurred separately within each of these farms.

### 3.4. Public Health Response and Recommendations

During the on-farm investigations, persons on all six farms received PPE kits containing N95 respirators, protective coveralls with hoods, gloves, and face shields; a training by public health officials to explain the reasons behind and importance of PPE use in their preferred language (English or Spanish); and a demonstration for safe donning and doffing and proper PPE disposal. The field team also applied a One Health approach to develop a mink farm investigation toolkit, including a farm-level and individual-level questionnaire, based on experiences conducting epidemiologic interviews with farm operators and workers to serve as a resource for systematic data collection in future investigations of SARS-CoV-2 on mink farms [40]. The toolkit was used by other state health departments for investigations and shared with federal One Health partners. Guidance and recommendations from CDC and USDA in June 2020, for preventing SARS-CoV-2 introduction and spread on mink farms [38,41] were explained to farm operators and to other workers if they were available. Farm workers were recommended to use eye protection (goggles or face shield), gloves, and boots and coveralls that were disinfected or laundered daily, and a fit-tested N95 respirator, particularly when mink were ill, or surgical mask, at a minimum. Workers at greater risk (e.g., persons aged ≥65 years, immunocompromised persons) were directed to not work in barns with mink known to be ill.

Farm operators were advised that any potentially ill workers should not work on-farm and should isolate according to CDC guidelines at the time. Farm operators were also advised to send home potentially ill workers who lived offsite, separate those that lived onsite from other residents, and to maintain a daily list of onsite workers for contact tracing if an ill worker was identified. However, most farms only had two to three total workers and often had no other feasible options to continue caring for the mink daily, even if they were feeling ill. Additionally, it was often not possible to restrict worker tasks to areas with sick mink or areas with healthy mink due to the small number of available workers. Once human vaccines became available in 2021, all persons working on mink farms were recommended to be vaccinated to help reduce the risk of introduction of SARS-CoV-2 from people to mink and other animals on-farm.

Operators of farms with symptomatic or SARS-CoV-2-positive mink were recommended to not share equipment or feed between barns and to assign specific workers to barns with ill mink if possible, or to have workers care for healthy animals before caring for sick animals. Due to the rapid spread of infection among mink and space constraints on farms in this investigation, farms were generally not able to isolate ill mink or quarantine exposed mink; however, all participating farms were recommended to feed healthy mink prior to feeding any ill mink to avoid contamination of food or feeding equipment and to not share workers or equipment across farms. Finally, farm operators were recommended to restrict visitors and other animals (e.g., rodents, birds, wildlife, and domestic animals) from the premises, maintain ≥6 feet distance between all people working on the farm, quarantine any new mink introduced to the farm, and clean and disinfect frequently touched surfaces such as door handles, counters, and control panels for feed delivery systems.

In November 2020, recommendations for preventing SARS-CoV-2 introduction and spread on mink farms were also presented in-person during a special session at a fur breeders conference in Utah where participants also received PPE kits and instructional handouts for PPE use and worker health screening practices.

## 4. Discussion

At the time of this report, the United States had confirmed SARS-CoV-2 infections in mink on 18 farms across five states: 12 in Utah, three in Wisconsin, one in Michigan, one in Oregon, and one state anonymized by state and federal animal health officials [23]. Following identification, affected farms with active SARS-CoV-2 infections were placed under immediate quarantine by the State Animal Health Official to restrict movement of animals, animal products, and animal waste on and off farm until state officials determined that SARS-CoV-2 was no longer circulating on the farms or posed a threat to public health [38]. These farms complied with quarantine regulations and followed the recommended guidelines for quarantine release established by USDA.

During 1 August–30 November 2020, incidence of confirmed human SARS-CoV-2 infections in Utah increased from about 400 cases per day to 2700 cases per day. In Utah, the location of family residences on-farm could have led to household or workplace transmission among associated human cases and may have subsequently contributed to elevated transmission rates among workers on participating farms.

The source of SARS-CoV-2 introduction to the affected mink herds was unknown prior to our investigations. We subsequently identified at least one human case of molecularly and/or serologically confirmed COVID-19 on each of the six participating farms. On five farms, a laboratory-confirmed COVID-19 case with known symptom onset and mink contact while symptomatic, prior to illness onset in mink, was identified as the presumed epidemiologic link on each associated farm. However, on one farm, Farm D, the one identified SARS-CoV-2 seropositive human case was a worker who did not report having any history of symptoms consistent with COVID-19 prior to illness onset in mink, so the epidemiologic link is less clear. Longitudinal sampling on two farms (Farms A and B) revealed persistent viral shedding in the mink herds. SARS-CoV-2 RNA was detected in specimens from mink at the final field team collection TPs on each farm, 70 and 116 days after initial onset of clinical signs in the mink herds, respectively. Notably, virus isolation was not successful from specimens obtained from mink at the third TPs, suggesting that while the mink may have had persistent detectable viral RNA, they might not have been infectious. Serological testing of mink revealed 100% seropositivity for SARS-CoV-2 that persisted until four- and two-months post-onset of herd mortality (in Farms A and B, respectively), with robust detectable immune response. These investigations also identified molecular and serologic evidence of SARS-CoV-2 infection in domestic animals (dogs and feral/free-roaming cats) on affected farms, but no evidence of SARS-CoV-2 in peridomestic wildlife trapped and sampled on the farms during these investigations. Moreover, the results obtained from three different serological assays (two virus neutralization assays conducted in two laboratories, and one mix-and-read assay) were in high agreement, further supporting the utility of the mix-and-read assay as a species-independent serologic tool.

Genomic analyses suggest that SARS-CoV-2 transmission occurred within each farm, rather than between farms. SARS-CoV-2 sequences generated from humans, cats, and dogs were almost identical to at least one sequence from mink on the same farm. Additionally, some investigation sequences contained Spike protein mutations previously detected in the human population. Together with the epidemiologic data, this suggests community-acquired SARS-CoV-2 infection in a human case(s) as the likely source of introduction to the mink herds, with subsequent transmission among mink and spillover into susceptible on-farm domestic animals. Notably, the cats tested during this investigation were feral/free-roaming with no known history of close contact with humans, so infected mink represent the most likely source of SARS-CoV-2 exposure to the cats. However, as a sub-study of these investigations, a subset of these sampled cats was fitted with GPS collars and released back on the farms where they were captured [29]. Results from GPS tracking of these cats showed they made frequent visits to mink sheds, moved freely around the affected farms, and visited surrounding residential properties and neighborhoods, and as such, exposure to SARS-CoV-2 off-farm cannot be ruled out [29]. In contrast, the dogs were housed on-farm, and had exposure to humans, including those with a history of COVID-19, so the source of SARS-CoV-2 infection in the dogs is less clear.

Aside from Y453F, a mutation which was identified in some mink and mink-associated sequences, no additional Cluster 5 mutations were present in any mink or mink farm-associated sequences. Additionally, sequences from longitudinal sampling on Farms A and B over a four- and two-month time span, respectively, had increased diversity, suggesting continued viral evolution within the population over time, though notably most of the sequences were still identical or varied by a couple of SNPs, consistent with an expected rate of viral evolution within a population over time [42].

The short time between initial exposure of mink to a human case and the first signs of illness in mink suggest that workers likely transmitted infection to mink early in their illness. Some workers were likely unaware of being infected, due to asymptomatic illness, being in the pre-symptomatic phase of illness, or having only mild symptoms. A few workers demonstrated rRT-PCR-positivity at multiple time points, but such periods have been observed elsewhere, are not necessarily associated with infectivity, and did not appear to affect the trajectories of the outbreak in mink on their farms [43,44].

Agricultural workers, designated as “essential” workers [45], continued to work throughout the COVID-19 pandemic and were at increased risk for contracting COVID-19 [46]. Mink farms present additional unique challenges for conducting public health investigations and introducing preventive interventions. In our study, the participating farms were small, family-operated farms with few workers and typically ≤2 hired employees. Workers who did not live on-farm (i.e., in the family home) typically shared housing in the community. Since the mink industry is not currently regulated by the U.S. Department of Agriculture Animal and Plant Health Inspection Service (USDA-APHIS, Riverdale, MD, USA), workplace and animal health policies and practices are generally not formalized. Additionally, some farm operators were hesitant to share information with the field team regarding infection control, biosecurity, or workplace practices for privacy and security reasons.

The findings here are subject to some limitations. First, this investigation emphasized SARS-CoV-2 infections in humans and mink on the farms. Other potential introductions of SARS-CoV-2 to farms were not assessed comprehensively and cannot be ruled out, although a presumptive human-to-mink transmission link was identified on each farm investigated and is the likely source of initial introduction to the herd. Additionally, the role that environmental contamination may have played in SARS-CoV-2 transmission on the farms was also not systematically assessed. However, viral RNA was detected from fur swabs collected from mink, dogs, and cats, suggesting extensive environmental contamination at the time of sampling, though cycle threshold values suggested that an infectious virus would not have been recovered from these specimens. Secondly, participants provided their best estimate of symptom onset dates; we interpreted the window of exposure to mink while the person was more broadly infectious (i.e., up to and on the first day of increased mink mortality) to capture all potential sources of human-to-mink transmission but the definitive direction of infection cannot be determined, especially as the actual onset of SARS-CoV-2 infection in mink was likely earlier than the first signs of illness or mortality in mink. Thirdly, because participation in the investigations was voluntary and several farms with confirmed SARS-CoV-2 in mink were unwilling to participate, this was a convenience sample of relatively small family farms and as such the transmission dynamics observed here may not apply to larger farms or those farms located in other states or countries. Finally, mitigation of the spread of infection or mortality in mink was not evaluated systematically. However, the provision of PPE kits and demonstrations established a new baseline for COVID-19 prevention education and practice on Utah mink farms.

Knowledge of SARS-CoV-2 infection in mink was limited at the time of these investigations, particularly in the United States. Although CDC guidance as early as January 2020 advised that people with COVID-19 who live or work with animals wear masks to prevent transmission to animals [47], and mink farm workers in European countries had been advised to wear masks as early as summer 2020, few workers in Utah were employing infection prevention protocols, such as face masks, when working with mink. Non-pharmaceutical interventions, such as staying home if experiencing symptoms or after testing positive, wearing masks and gloves, and cleaning and disinfecting surfaces, are imperative for COVID-19 prevention in people and susceptible animal species in farm environments for many reasons, including the mobility of transient workers (e.g., temporary laborers, providers of equipment, supplies, and feed) on and off the farm, the high likelihood of indoor environmental contamination, the potential for infection among a variety of animals including livestock, and the possibility of transmission between people and animals [10,21,48,49].

From December 2020 through August 2022, there were no new reports of farms in the United States with clinical SARS-CoV-2 infections in farmed mink. Human COVID-19 vaccines became available in December 2020. While early studies suggested that individuals living in rural areas or working in the agricultural sector may have increased vaccine hesitancy, there are no specific data available on vaccine uptake among mink farm workers [46,50,51]. Additionally, USDA-APHIS approved an experimental SARS-CoV-2 vaccine for mink, allowing U.S. farms access to preventative vaccination for farmed mink beginning in May 2021 [52]. The introduction of SARS-CoV-2 vaccines in humans and mink, in combination with the rapid development and distribution of infection prevention guidance by CDC, UDAF, UDHHS, and USDA-APHIS to mink farms nationwide, may have helped to prevent further outbreaks. However, in the absence of active surveillance for SARS-CoV-2 on mink farms in the United States, it is impossible to know the true incidence of mild or asymptomatic SARS-CoV-2 infection in farmed mink, representing a gap in U.S. surveillance capacity for monitoring SARS-CoV-2 viral evolution and the emergence of new mutations or variants with potential public health impacts. Additionally, more research is needed to investigate animal SARS-CoV-2 vaccine effectiveness against novel SARS-CoV-2 variants as they continue to emerge.

## 5. Conclusions

Our findings underline the importance of taking a One Health approach when applying preventive measures in mink farm environments to prevent the introduction of SARS-CoV-2 from people to animals and to stop the further spread of the virus among animals and between animals and people. In our sample of six affected Utah farms that participated in on-farm investigations, SARS-CoV-2 spread rapidly among mink shortly after they were first exposed. Illness and mortality among the mink herds could not be mitigated, leading to extensive loss and economic burden to producers, with some farms losing up to 50% of their herds. Interestingly, the high mortality experienced in these farms differed from the asymptomatic or mild infections described in most European farms. This is likely related to the active surveillance and testing strategy for SARS-CoV-2 in mink farms in Europe, in contrast to the United States, where testing typically has occurred only after identification of severe disease in mink, and farms with only asymptomatic or mild infection may not have been identified. Domestic surveillance is also complicated by the fact that mink farming in the United States is not a regulated livestock sector by the USDA or most state departments of agriculture. There is no mechanism for indemnity for economic losses, and as a result there is little incentive for producers to voluntarily report suspect cases of SARS-CoV-2 infection in mink herds.

Moving forward, a One Health approach engaging local, subnational, national, and global public health, agriculture, wildlife, environment officials, and industry partners is critical to effectively address SARS-CoV-2 and other emerging infectious diseases in farmed mink and other animal species. Our study suggests that outreach and education regarding preventive measures within communities and populations associated with farm environments where susceptible animals, including mink, are raised, is a central component of a One Health approach and is crucial to preventing the introduction and spread of SARS-CoV-2 to people and mink. Active surveillance and further research of SARS-CoV-2 in mink and other susceptible animals is critical to monitor viral evolution, to detect potential emerging variants of concern, and to better understand the public and animal health risk for zoonotic transmission of SARS-CoV-2 in the United States and globally.

## Figures and Tables

**Table 1:** Estimated mink herd sizes prior to infection and losses due to SARS-CoV-2 by farm (as reported by farm operators).

Farm	Est. Mink Herd Size Prior to Losses	Est. Mink Losses due to SARS-CoV-2 (%)
A	7000	1454 (19.9)
B	8000	1251 (15.2)
C	1500	65 (4.3)
D	600	300 (50.0)
E	13,500	2000 (14.8)
F	300	150 (50.0)

**Table 2:** Summary of longitudinal animal SARS-CoV-2 rRT-PCR and serology results by farm and species.

	TP 1 *^,^ **			TP 2 *			TP 3 *^,^ **		
	No. rRT-PCR Positive/No. Tested (%)	No. Seropositive/No. Tested (%)	No. Positive by Either Test/No. Tested (%)	No. rRT-PCR Positive/No. Tested (%)	No. Seropositive/No. Tested (%)	No. Positive by Either Test/No. Tested (%)	No. rRT-PCR Positive/No. Tested (%)	No. Seropositive/No. Tested (%)	No. Positive by Either Test/No. Tested (%)
Farm A									
Dogs ^†^	1/1 (100)	1/1 (100)	1/1 (100)	0/1 (0)	1/1 (100)	1/1 (100)	0/1 (0)	1/1 (100)	1/1 (100)
Feral cats ^‡^	4/16 (25.0)	7/15 (46.7)	8/16 (50.0)	3/19 (15.8)	4/17 (23.5)	5/19 (26.3)	0/21 (0)	7/21 (33.0)	7/21 (33.0)
Mink	171/255 (67.0)	59/59 (100)	59/59 (100)	90/129 (70.0)	30/30 (100)	30/30 (100)	4/138 (2.9)	30/30 (100)	30/30 (100)
Farm B									
Dogs ^‡^	2/4 (50)	4/4 (100)	4/4 (100)	0/4 (0)	4/4 (100)	4/4 (100)	NA	NA	NA
Mink	33/60 (52)	61/61 (100)	61/61 (100)	31/31 (100)	31/31 (100)	31/31 (100)	NA	NA	NA

* TP = time point; TP 1 20–26 August; TP 2 9–13 October; TP 3 = 2–5 December. ** *Mus musculus* (rodents) (N = 22 on Farm A and N = 4 on Farm B) were also sampled; all were negative on SARS-CoV-2 rRT-PCR; one skunk on Farm A had only blood collected (no swabs) during TP3 and was seronegative. **^†^** The same dog(s) were sampled at each TP on Farms A and B. ^‡^ Cats were not microchipped in August. Cats in October were microchipped and two of those cats (one rRT-PCR positive and one negative) were resampled in December.

**Table 3:** Summary of animal SARS-CoV-2 rRT-PCR and serology results by farm and species in four Utah farms (Farms C to F) that experienced increased mortality in their mink herds beginning 10–29 September and which participated in field investigations from 2–10 October 2020.

	No. rRT-PCR Positive/No. Tested (%)	No. Seropositive/No. Tested (%)	No. Positive by Either test/No. Tested (%)
Farm C *			
Dogs	1/2 (50)	0/2 (0)	1/2 (50)
Feral cats	0/11 (0)	0/11 (0)	0/11 (0)
Mink	NT **	NT **	NT **
Farm D *			
Feral cats	1/5 (20)	4/4 (100)	4/5 (80)
Mink	2/2 (100)	NT **	2/2 (100)
Farm E *			
Dogs	1/2 (50)	1/2 (50)	1/2 (50)
Feral cats	4/7 (57)	3/4 (75)	4/7 (57)
Mink	29/31 (93.5)	1/1 (100) **	29/31 (93.5)
Farm F			
Mink	10/10 (100)	NT	10/10 (100)

* Wildlife sampling was all negative (1 raccoon (*Procyon lotor*) on Farm E; 1 raccoon and 1 skunk (*Mephitis mephitis*) on Farm C; 1 skunk on Farm D. ** NT = none tested as part of the described investigations; blood collected from one euthanized mink on Farm E.

**Table 4:** Phylogenetic analysis of sequences derived from human and animal specimens from six mink farms in Utah.

Farm	No. Human Sequences	No. Mink Sequences	No. Cat Sequences	No. Dog Sequences	NextstrainClade	SNP Differences	Detected Mutations in Spike Gene *
A	1	49	1	0	20C	0–7	Y453F
B	1	13	NT ^†^	0	20C	0–9	
C	0	3	0	0	20A	2–3	Y453F
D	0	4	0	NT ^†^	20A	0–2	Y453F
E	1	25	1	0	20A	0–8	Y453F
F	1	13	NT ^†^	NT ^†^	20A	0–3	Y453F

* All sequences contain the D614G mutation, and mink-associated “Cluster 5” mutations include H69-/V70-, Y453F, I692V, S1147L, M1229I. Among these mutations, only Y453F was detected in these investigation sequences. ^†^ NT = None tested as part of the described investigations.

## Data Availability

Complete or near-complete genome sequences of SARS-CoV-2 obtained in this investigation are available at Global Initiative on Sharing All Influenza Data (GISAID) and GenBank. Accession numbers and metadata for these sequences are available in Table S1. Additional information or de-identified data may be made available to researchers who submit a methodologically sound proposal to the corresponding author.

## Supplementary Materials

The following supporting information can be downloaded at: https://www.mdpi.com/article/10.3390/v15010096/s1, Figure S1: Epidemiological timeline, evidence of SARS-CoV-2 infection among 16 persons on six mink farms—Utah, 30 July–15 October 2020; Figure S2: Virus neutralization and agreement between different serology assays. Qualitative agreement between assay pairs was assessed by calculating the kappa statistic using online version of GraphPad at https://www.graphpad.com/quickcalcs/kappa1.cfm (accessed on 1 November 2022) which calculated confidence intervals with equations by Fleiss. For the purposes of this analysis, the suspect results from the CPE-based assay (1/16 serum dilution) were considered negative. For presentation purposes, the specimens in the figure on the right who tested negative by both serology assays are nudged to reveal otherwise overlapping data points; Table S1: Accession numbers and metadata for 113 SARS-CoV-2 sequences from mink and mink-associated host (human, cat) specimens from six mink farms in Utah, including some sequences previously published in Eckstrand et al., 2021 [39].

## Appendix A. Animal Sample Collection and Sampling Strategy

Longitudinal sampling was conducted in mink herds on two farms (A and B). Mink on both farms were collected by UDAF on August 10 for initial diagnosis following reports of increased mortality. Field investigations began the week following the initial confirmation by SARS-CoV-2 rRT-PCR at USDA-NVSL on August 17 [23]. On Farm A, longitudinal sampling of the mink herd, feral/free-roaming cat colony, and one dog was conducted at three time points (TPs) in August, October, and December (e.g., 1–2 weeks, 8–9 weeks, and 16 weeks after onset of increased mink mortality, respectively). On Farm B, longitudinal sampling of the mink herd and four farm dogs occurred at two TPs in August and October (e.g., 3.5–4 weeks, 8–9 weeks after onset of increased mink mortality). Location, age, sex, and presence of clinical signs consistent with SARS-CoV-2 infection, or any observed significant medical abnormality, were noted for each animal.

On Farm A, non-invasive specimens (oropharyngeal [OP] and rectal swabs) were collected from randomly sampled live, unsedated mink by restraining the animals behind the shoulders and at the base of the tail. A sterile 1-mL syringe was placed at the commissure of the mouth to allow for collection of an OP swab by sliding the swab over the syringe to the oropharynx, taking care to avoid the tongue and saliva contamination. Swabs were collected using plastic-handled polyester-tipped swabs which were cut with clean scissors before placing into a sterile 2 mL tube containing 0.5 mL viral transport medium (VTM). Smaller numbers of mink were randomly selected for terminal sampling on Farms A and B at each TP. Notably, mink on Farm A were randomly selected regardless of presence of clinical signs, while mink on Farm B were selected largely based on presence of clinical signs (e.g., respiratory, neurological, or non-specific). Additionally, convenience sampled fresh dead mink (e.g., mink that succumbed to disease naturally and were found by farm workers in the morning) were also collected for testing on both farms.

On Farm A, non-destructively sampled feral cats were trapped with live traps (36″L × 10″W × 12″H; Tomahawk, Hazelhurst, WI, USA). Cats were sedated using 3–5 mg/kg ketamine and 0.005–0.02 mg/kg dexmedetomidine injected intramuscularly (IM). Inhalant isoflurane was used as needed to maintain sedation. Following specimen collection in the cats, dexmedetomidine was reversed with an equal volume of atipamezole injected IM. Additionally, terminally sampled mink and trapped wildlife were sedated prior to sampling and euthanasia with 0.03–0.04 mg/kg dexmedetomidine and 5 mg/kg ketamine injected IM. After sedation had taken effect, mink were removed from cages, and cats and wildlife from traps, and taken to a clean processing area for specimen collection. OP, NP, and rectal swabs were collected from sedated mink, cats, and wildlife, and from dogs without use of sedation. Fur swabs were also collected in August and October from a subset of animals on participating farms to assess environmental contamination with SARS-CoV-2. OP, rectal, and fur swabs were collected using sterile plastic-handled polyester-tipped swabs which were cut with clean scissors before placing into a sterile 2 mL tube containing 0.5 mL VTM. NP swabs were collected using sterile metal-handled mini polyester-tipped swabs which were cut with clean scissors before placing into a sterile 2 mL tube containing 0.5 mL VTM.

Up to 3 mL of blood was collected from dogs via the cephalic or lateral saphenous veins and from sedated cats via the jugular or medial saphenous veins. On terminally sampled animals, up to 3 mL of intracardiac (IC) blood was collected after sedation. Subsequently, for terminally sampled mink and wildlife, sodium pentobarbital 0.05 mg/kg was injected IC for euthanasia. Terminally sampled animals were placed in individual plastic bags with identifiers and placed in a cooler on ice until necropsy.

Rodents (*Mus musculus*) were also trapped on Farms A and B using H.B. Sherman folding live rodent traps (Tallahassee, FL) and subsequently euthanized using isoflurane at TP1. OP and rectal swabs were collected from rodents as described above and intracardiac blood was collected on necropsy.

Convenience fresh dead mink, up to five per day, dogs, and feral/free-roaming cats, were sampled, as described above, on four additional farms that began experiencing increased mortality in their mink herds in mid-September (Farms C–F). Terminally sampled wildlife (raccoon, skunks) on Farms C–F were trapped and specimens collected as described above.

After collection, swab specimen tubes were placed directly into liquid nitrogen for storage until transportation on dry ice to storage at −80 °C until testing. Blood specimens were centrifuged, and sera were removed, aliquoted, and stored frozen at −20 °C until transportation on dry ice for serological testing. On necropsy of euthanized animals, tissues were collected and stored in liquid nitrogen until transportation on dry ice to the National Wildlife Health Center (Madison, WI, USA) for eventual testing. Additional tissues were collected, fixed in formalin, and shipped to CDC for eventual histopathological analysis and SARS-CoV-2 immunohistochemistry.

Results from histopathological analysis, SARS-CoV-2 and other testing on tissue specimens are published separately [53,54]. Additionally, a sub-study by our team during the October field investigations placed collars with global positioning systems (GPS) on a subset of free-roaming cats on Farms A, C, and D to monitor movement patterns on- and off-farm; the findings from this sub-study are published separately [29].
