# Supplementary material for: One Health Investigation of SARS-CoV-2 in People and Animals on Multiple Mink Farms in Utah
Source: Viruses. 2022 Dec 29;15(1):96. doi: 10.3390/v15010096 (PMC9864593; doi:10.3390/v15010096)
Supplement: Supplementary file 1 [file viruses-15-00096-s001.zip › viruses-2061693-supplementary.pdf]

Supplementary Materials: The following supporting information can be downloaded at: www.mdpi.com/xxx/s1

Figure S1. Epidemiological timeline, evidence of SARS-CoV-2 infection among 16 persons on six mink farms — Utah, July 30–October 15, 2020[1,2]

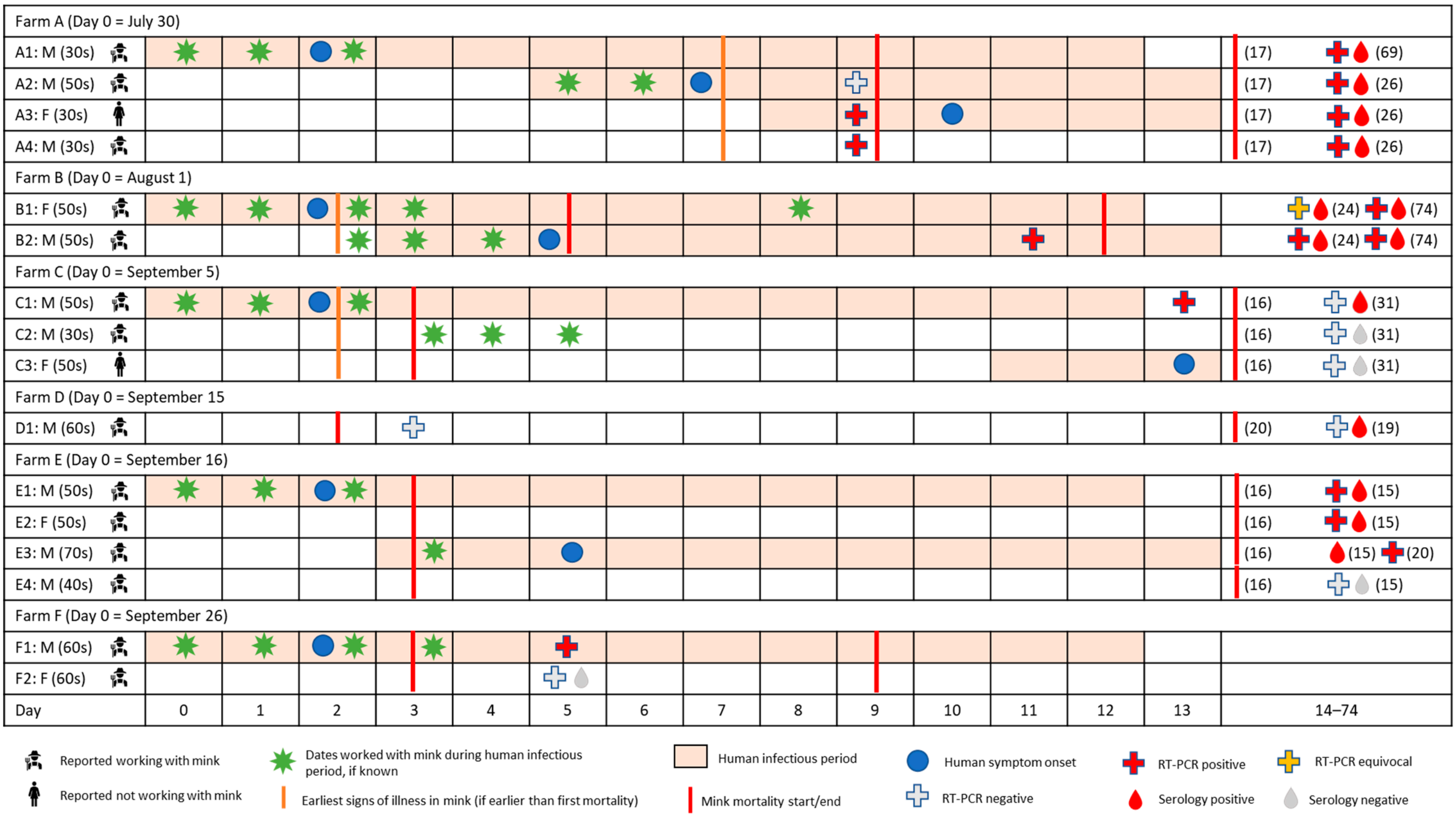

[1] Abbreviations: M=male; F=female; (30s)…(70s)=age of patient; (14)…(74)=number of days after the beginning of infectious period for the first human case associated with each farm that mink mortality ended or test result obtained

[2] Human infectious period was defined as 2 days before symptom onset (or 2 days prior to positive sample collection in asymptomatic persons) up to 10 days after symptom onset.

Figure S2. Virus neutralization and agreement between different serology assays. Qualitative agreement between assay pairs was assessed by calculating the kappa statistic using online version of GraphPad at https://www.graphpad.com/quickcalcs/kappa1.cfm, which calculated confidence intervals with equations by Fleiss. For the purposes of this analysis, the suspect results from the CPE-based assay (1/16 serum dilution) were considered negative. For presentation purposes, the specimens in the figure on the right that tested negative by both serology assays are nudged to reveal otherwise overlapping data points.

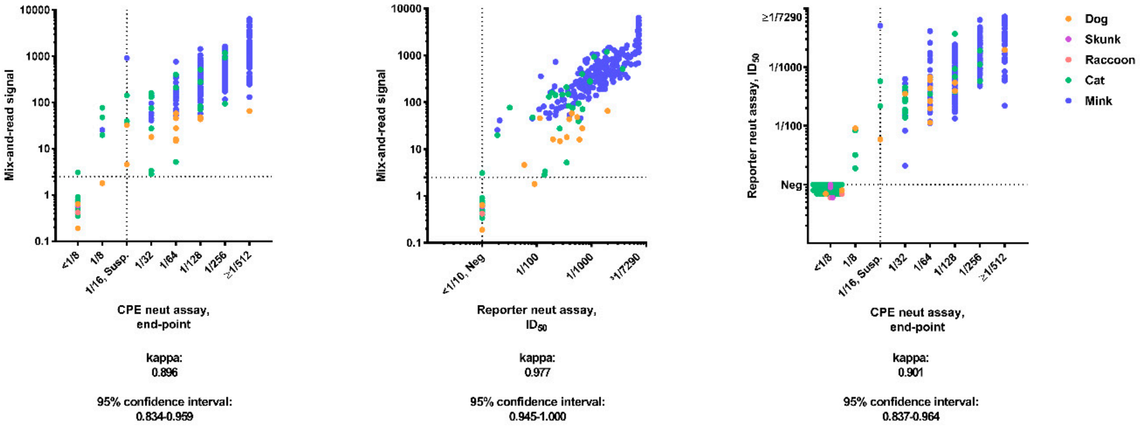

Table S1. Accession numbers and metadata for 113 SARS-CoV-2 sequences from mink and mink-associated host (human, cat) specimens from six mink farms in Utah, including some sequences previously published in Eckstrand et al, 2021 [39].

gisaid_epi_isl	Host species	Collection date	Farm ID
EPI_ISL_10026508	*Neogale vison*	10/4/2020	D
EPI_ISL_10026513	*Neogale vison*	8/22/2020	A
EPI_ISL_2834689	*Neogale vison*	10/1/2020	A
EPI_ISL_2834690	*Neogale vison*	10/1/2020	A
EPI_ISL_2834939	*Neogale vison*	9/14/2020	A
EPI_ISL_2834940	*Neogale vison*	9/14/2020	A
EPI_ISL_2834941	*Neogale vison*	9/14/2020	A
EPI_ISL_2834942	*Neogale vison*	9/22/2020	A
EPI_ISL_2834943	*Neogale vison*	9/22/2020	A
EPI_ISL_2834944	*Neogale vison*	9/22/2020	A
EPI_ISL_2834945	*Neogale vison*	9/22/2020	A
EPI_ISL_2896203	*Neogale vison*	8/10/2020	A
EPI_ISL_2896204	*Neogale vison*	8/10/2020	A
EPI_ISL_2896205	*Neogale vison*	8/10/2020	A
EPI_ISL_2896206	*Neogale vison*	8/10/2020	A
EPI_ISL_2896207	*Neogale vison*	8/14/2020	A
EPI_ISL_2896208	*Neogale vison*	8/14/2020	A
EPI_ISL_594062	Human	10/1/2020	A
EPI_ISL_812134.2	Human	10/1/2020	E
EPI_ISL_812135.2	Human	8/22/2020	B
EPI_ISL_9936844	*Neogale vison*	8/20/2020	B
EPI_ISL_9936845	*Felis catus*	10/2/2020	E
EPI_ISL_9936846	*Neogale vison*	10/2/2020	E
EPI_ISL_9936847	*Neogale vison*	10/4/2020	E
EPI_ISL_9936848	*Neogale vison*	10/4/2020	E
EPI_ISL_9936849	*Neogale vison*	8/25/2020	A
EPI_ISL_9936850	*Neogale vison*	8/25/2020	A
EPI_ISL_9936851	*Neogale vison*	8/25/2020	A
EPI_ISL_9936852	*Felis catus*	8/26/2020	A
EPI_ISL_9936853	*Neogale vison*	8/22/2020	A
EPI_ISL_9936854	*Neogale vison*	8/22/2020	A
EPI_ISL_9936855	*Neogale vison*	8/22/2020	A
EPI_ISL_9936856	*Neogale vison*	8/23/2020	A
EPI_ISL_9936857	*Neogale vison*	8/24/2020	A
EPI_ISL_9936858	*Neogale vison*	8/25/2020	A
EPI_ISL_9936859	*Neogale vison*	8/23/2020	A
EPI_ISL_9936860	*Neogale vison*	8/20/2020	A
EPI_ISL_9936861	*Neogale vison*	8/20/2020	B

EPI_ISL_9936862	*Neogale vison*	8/24/2020	A
EPI_ISL_9936863	*Neogale vison*	8/24/2020	A
EPI_ISL_9936864	*Neogale vison*	8/24/2020	A
EPI_ISL_9936865	*Neogale vison*	8/29/2020	B
EPI_ISL_9936866	*Neogale vison*	10/2/2020	E
EPI_ISL_9936869	*Neogale vison*	10/2/2020	E
EPI_ISL_9936872	*Neogale vison*	10/2/2020	E
EPI_ISL_9936873	*Neogale vison*	10/2/2020	E
EPI_ISL_9936874	*Neogale vison*	10/2/2020	E
EPI_ISL_9936875	*Neogale vison*	10/2/2020	F
EPI_ISL_9936877	*Neogale vison*	10/2/2020	F
EPI_ISL_9936878	*Neogale vison*	10/2/2020	F
EPI_ISL_9936879	*Neogale vison*	10/2/2020	F
EPI_ISL_9936882	*Neogale vison*	10/2/2020	F
EPI_ISL_9936884	*Neogale vison*	10/3/2020	E
EPI_ISL_9936886	*Neogale vison*	10/3/2020	E
EPI_ISL_9936887	*Neogale vison*	10/3/2020	E
EPI_ISL_9936890	*Neogale vison*	10/3/2020	E
EPI_ISL_9936892	*Neogale vison*	10/3/2020	E
EPI_ISL_9936893	*Neogale vison*	10/3/2020	F
EPI_ISL_9936895	*Neogale vison*	10/3/2020	F
EPI_ISL_9936897	*Neogale vison*	10/3/2020	F
EPI_ISL_9936900	*Neogale vison*	10/3/2020	F
EPI_ISL_9936902	*Neogale vison*	10/3/2020	F
EPI_ISL_9936904	*Neogale vison*	10/4/2020	E
EPI_ISL_9936905	*Neogale vison*	10/4/2020	E
EPI_ISL_9936907	*Neogale vison*	10/4/2020	E
EPI_ISL_9936909	*Neogale vison*	10/6/2020	E
EPI_ISL_9936910	*Neogale vison*	10/6/2020	E
EPI_ISL_9936913	*Neogale vison*	10/6/2020	E
EPI_ISL_9936914	*Neogale vison*	10/8/2020	D
EPI_ISL_9936916	*Neogale vison*	10/8/2020	E
EPI_ISL_9936919	*Neogale vison*	10/8/2020	E
EPI_ISL_9936920	*Neogale vison*	10/9/2020	A
EPI_ISL_9936921	*Neogale vison*	10/9/2020	A
EPI_ISL_9936922	*Neogale vison*	10/9/2020	A
EPI_ISL_9936923	*Neogale vison*	10/9/2020	A
EPI_ISL_9936924	*Neogale vison*	10/9/2020	A
EPI_ISL_9936925	*Neogale vison*	10/9/2020	A
EPI_ISL_9936926	*Neogale vison*	10/9/2020	A
EPI_ISL_9936927	*Neogale vison*	10/9/2020	A
EPI_ISL_9936928	*Neogale vison*	10/9/2020	A
EPI_ISL_9936929	*Neogale vison*	10/9/2020	A
EPI_ISL_9936930	*Neogale vison*	10/9/2020	A
EPI_ISL_9936931	*Neogale vison*	10/9/2020	A
EPI_ISL_9936932	*Neogale vison*	10/9/2020	A
EPI_ISL_9936933	*Neogale vison*	10/9/2020	A
EPI_ISL_9936934	*Neogale vison*	10/9/2020	A
EPI_ISL_9936935	*Neogale vison*	10/9/2020	A
EPI_ISL_9936936	*Neogale vison*	10/9/2020	A
EPI_ISL_9936937	*Neogale vison*	10/9/2020	A
EPI_ISL_9936938	*Neogale vison*	10/9/2020	A
EPI_ISL_9936939	*Neogale vison*	10/9/2020	A
EPI_ISL_9936940	*Neogale vison*	10/9/2020	A
EPI_ISL_9936941	*Neogale vison*	10/9/2020	A
EPI_ISL_9936942	*Neogale vison*	10/10/2020	A
EPI_ISL_9936944	*Neogale vison*	10/10/2020	A
EPI_ISL_9936945	*Neogale vison*	10/10/2020	A
EPI_ISL_9936946	*Neogale vison*	10/11/2020	A
EPI_ISL_9936948	*Neogale vison*	10/11/2020	A
EPI_ISL_9936949	*Neogale vison*	10/11/2020	A
EPI_ISL_9936950	*Neogale vison*	10/11/2020	A
EPI_ISL_9936951	*Neogale vison*	10/11/2020	A
EPI_ISL_9936953	*Neogale vison*	10/12/2020	B

EPI_ISL_9936956	*Neogale vison*	10/12/2020	B
EPI_ISL_9936957	*Neogale vison*	10/12/2020	B
EPI_ISL_9936958	*Neogale vison*	10/12/2020	B
EPI_ISL_9936959	*Neogale vison*	10/13/2020	B
EPI_ISL_9936961	*Neogale vison*	10/13/2020	B
EPI_ISL_9936962	*Neogale vison*	10/13/2020	B
EPI_ISL_9936963	*Neogale vison*	10/13/2020	B
EPI_ISL_9936964	*Neogale vison*	10/2/2020	E
EPI_ISL_9936965	*Neogale vison*	10/3/2020	E
EPI_ISL_9936966	*Neogale vison*	10/3/2020	F
EPI_ISL_9936967	*Neogale vison*	10/6/2020	E
